# Rice powder template for hausmannite Mn_3_O_4_ nanoparticles and its application to aqueous zinc ion battery

**DOI:** 10.1371/journal.pone.0305611

**Published:** 2024-06-17

**Authors:** Nusrat Tazeen Tonu, Parbhej Ahamed, Mohammad Abu Yousuf

**Affiliations:** 1 Department of Chemistry, Khulna University of Engineering & Technology, Khulna, Bangladesh; 2 Chemistry Discipline, Khulna University, Khulna, Bangladesh; Galala University, EGYPT

## Abstract

In this study, a simple calcination route was adopted to prepare hausmannite Mn_3_O_4_ nanoparticles using rice powder as soft bio-template. Prepared Mn_3_O_4_ was characterized by Fourier Transform Infra-Red Spectroscopy (FTIR), Field Emission Scanning Electron Microscopy (FESEM), Energy Dispersive X-ray microanalysis (EDX), Powder X-Ray Diffraction (XRD), Transmission Electron Microscopy (TEM), Brunauer-Emmett-Teller (BET) and Solid state UV-Vis spectroscopic techniques. Mn-O stretching in tetrahedral site was confirmed by FTIR and Raman spectra. % of Mn and O content supported Mn_3_O_4_ formation. The crystallinity and grain size was found to be 68.76% and 16.43 nm, respectively; tetragonal crystal system was also cleared by XRD. TEM clarified the planes of crystal formed which supported the XRD results and BET demonstrated mesoporous nature of prepared Mn_3_O_4_ having low pore volume. Low optical band gap of 3.24 eV of prepared Mn_3_O_4_ nanoparticles indicated semiconductor property and was used as cathode material to fabricate CR-2032 coin cell of Aqueous Rechargeable Zinc Ion Battery (ARZIB). A reversible cyclic voltammogram (CV) showed good zinc ion storage performance. Low cell resistance was confirmed by Electrochemical Impedance Spectroscopy (EIS). The coin cell delivered high specific discharge capacity of 240.75 mAhg^-1^ at 0.1 Ag^-1^ current density. The coulombic efficiency was found to be 99.98%. It also delivered excellent capacity retention 94.45% and 64.81% after 300 and 1000 charge-discharge cycles, respectively. This work offers a facile and cost effective approach for preparing cathode material of ARZIBs.

## Introduction

Safe, environment friendly, and cost-effective energy storage systems are ideal for large-scale application. Due to increasing urgency of tackling climate change and ecological deterioration, electrochemical energy storage systems are in high demand [1]. Decades have passed since rechargeable lithium-ion batteries dominated energy storage in portable devices and electric vehicles. But, few issues like, safety and eco-friendly stuff, expensive scaling up, and lack of lithium sources are all getting in the way of making big storage for the grid [2]. Lead-acid batteries (LABs) are the most used rechargeable batteries till now. It comprises about 60% of the world’s electric storage batteries [3]. Lead itself is toxic, and after the shelf life the Pb contaminated acid is thrown to the environment in most of the cases. During recycling a significant amount of poisonous Pb and As metals also go in the environment. Moreover, LABs has low energy-efficiency and short lifespan [4]. As opposed to the problems of the above mentioned rechargeable batteries aqueous rechargeable batteries based on Zn^2+^, Mg^2+^, Al^3+^, or Fe^3+^ ions are gaining popularity for low cost, superior conductivity, safety, ease of production, and environmental friendliness. Among them ARZIBs have gained significant consideration to the researchers. ARZIBs use zinc metal anodes, aqueous electrolytes, and different cathodes [5, 6]. Zn anode offers abundant sources, a theoretical high capacity of 820 mAhg^-1^, multivalent charge transport, adequate reduction potential (-0.76 V vs Standard hydrogen electrode), suitable ionic radii of Zn^2+^ ion (0.74 Å), and easy handling. In addition, aqueous electrolytes offer stronger conductivity, less dangers, and more safety. In ARZIBs, Zn anode is fixed but the cathode is different depending on the utilities [7, 8]. Prussian blue analogues (PBAs), manganese, vanadium, and organic species have been used to make ZIB cathodes. PBAs have a high potential (1.6–1.8V) but poor capacity (<100 mAhg^−1^) [9]. Although V-based cathode materials delivers high capacity and long cycling rate of performance, but they are costly, poisonous, and have a low voltage of operation (0.6–0.9 V). Likewise, cathode materials having organic framework have high value of specific capacity, but poor conductivity and low operating voltage [10, 11]. On the contrary, Mn-based cathode materials, with polymorphs or different crystal structure (α-, β-, δ-, γ-, λ-, T-type, R-type, ε- MnO_2_) and oxidation states (MnO, Mn_2_O_3,_ and Mn_3_O_4_), have gained the most attention of the researchers for their high specific capacity (308 mAhg^-1^), cost effectiveness, non toxicity, and high operating voltage (1.2–1.5 V) than Zn anodes [12, 13].

As a promising candidate, Mn_3_O_4_, having chemical formula Mn(II)Mn(III)_2_O_4_, has high capacity and voltage. In real ZIBs, these manganese oxides always have low conductivity and high volume expansion during cycling, causing electrode pulverization, structural collapse, and quick capacity fading. To obtain high-rate and long-life ZIBs, Mn_3_O_4_ needs innovative design methodologies [14, 15]. Spinel structured Mn_3_O_4_ is known for its thermodynamic stability, which helps prevent structural collapse during the electrode reaction process as well as impressive charging-discharging cycling stability and good capacity retention [16]. The distinctive and unwavering hausmannite structure exhibited by Mn_3_O_4_ at ambient temperature facilitates the production process of phase-pure Mn_3_O_4_ nanocrystals. These factors provide significant advantages to Mn_3_O_4_ as electrode materials for energy storage. Nevertheless, the practical implementation of Mn_3_O_4_ is constrained by its low active surface area, contemptable electronic conductivity, and vulnerability of accumulation during the synthesis route [17]. These disadvantages of Mn_3_O_4_ also limit its practical application in electrochemical energy storage systems. Several methods have been adopted to improve its electrochemical performance, such as- particle size reduction, uniform distribution of particles, and/or creating a mesoporous structure [18, 19]. Zhang *et al*. have effectively synthesized a diverse array of Mn_3_O_4_ nanorods featuring various nanostructures and microstructures. At applied current density of 0.1 Ag^-1^, the Mn_3_O_4_ electrode showed a specific capacitance of 136.5 Fg^-1^ [20]. In 1.0 M Na_2_SO_4_, Liu *et al*. synthesized solid nano-spheres of Mn_3_O_4_ which showed a specific capacitance of 150 Fg^-1^ at 0.3 Ag^-1^ current density [21]. In order to fabricate the Co(OH)_2_/Mn_3_O_4_ nanocomposites, Liu *et al*. utilized a facile ion diffusion technique to inject Co ions into Mn_3_O_4_. This modification significantly enhanced the aggregation characteristics of the Mn_3_O_4_ nanoparticles, as well as their conductivity and specific capacitance [22].

The method of using template for producing nanomaterials offers synthetic materials with controllable morphology, structure, and size with a high repetition rate. Rice powder being a non-metallic soft template is suitable for metal oxide nanoparticle synthesis [23]. In rice, starch is the main component. Starch consists carbohydrate polymeric chains which are build up from small glucose molecules and are parted in amylase (linear) and amylopectin (branched) units. These specific distinct characteristics of rice are the basic foundations for the synthesis of nanomaterials of different sizes and shapes [24]. Therefore, using rice powder as a sift bio template seem to be a promising way to synthesize manganese based oxide nanoparticles.

Lots of experiments have been done for the synthesis of various functional materials by using rice husk [23, 25] and starches [26, 27]. However, to the best of our knowledge, no such study has been found in the open literature for the synthesis of Mn_3_O_4_ nanoparticles using rice powder as soft bio template. Keeping in mind, in our study, rice powder was chosen as a soft template for the preparation of Mn_3_O_4_ nanoparticles due to the reasons- (i) its high porous structure, (ii) spongy character with water, (iii) special components having thermal degradation property, (iv) cost effective, and (v) available in local market. We hope this study will contribute some knowledge worth to be carried out. The prepared Mn_3_O_4_ has been characterized by FTIR, Raman, FE-SEM, EDX, TEM, BET, solid state UV- Visible spectroscopy and powder XRD techniques. Results showed nano sized Mn_3_O_4_ particle having low band gap and that was used as cathode material to fabricate CR-2032 coil cell of ARZIBs. Then the fabricated CR-2032 coil cell has been tested by CV, Battery Charging-Discharging (BCD) profile and EIS techniques to assess its electrochemical performance for reversible zinc ion storage.

## Materials and methods

### Chemicals and instruments

Manganese (II) acetate tetrahydrate, (CH_3_COO)_2_Mn.4H_2_O, was purchased from Sigma-Aldrich (USA). Rice powder was purchased from local stationary shop (Bangladesh). Stainless steel (SS) substrates (304 grades) of thickness t-0.01× w-100mm, Zinc foil, polyvinylidene fluoride (PVDF), n-methyl pyrrolidone (NMP), carbon black (C-black) were used for the preparation of the CR-2032 coin cell battery. The whole experimental was carried out using deionized (DI) water.

The structure and morphology of prepared Mn_3_O_4_ was investigated using FE-SEM machine (JSM-7610F, Japan). XRD analysis was done using X-ray diffractometer (XRD; Bruker, D2 PSASER) with Cu Kα radiation at 2θ from 10° to 80°. TEM analysis was done by JEOL TEM (Model: JEM 2100 PLUS, JEOL, Japan). Few amount of prepared sample was taken in a test tube containing ethanol and was shaken well. It was covered by a cork and sonicated for 30 min. Then the tube was kept at rest for a day. A drop of the sonicated sample was taken in a copper grid and kept in a vacuum chamber overnight for degassing. Then the copper grid contain sample was tested by TEM analyzer. BET was carried out using BET Sorptometer (Model: BET-201-A, ID: CRF-FR.B.02, PMI, USA). The prepared sample was pretreated with degassing at 90°C for 3 h followed by 20 microns vacuum at 130°C overnight in an oven with high purity nitrogen purge. Then it was analyzed by BET analyzer with respect to the adsorption/desorption of nitrogen. The thermal stability of rice powder was investigated using TGA machine (TGA-50, Shimadzu, Japan), from room temperature to 800°C (10°C/min, 2mL/min Air, Pt pan). To examine the semiconductor behavior of prepared Mn_3_O_4_, solid state UV-visible spectroscopic test was carried out within the range 300–800 nm using UV-1800 spectrophotometer of Shimadzu Corporation, Japan. CV, EIS and BCD were performed via Biologic (SP-300) potentiostat.

### Preparation of Mn_3_O_4_ using rice powder as template

**Fig 1:**
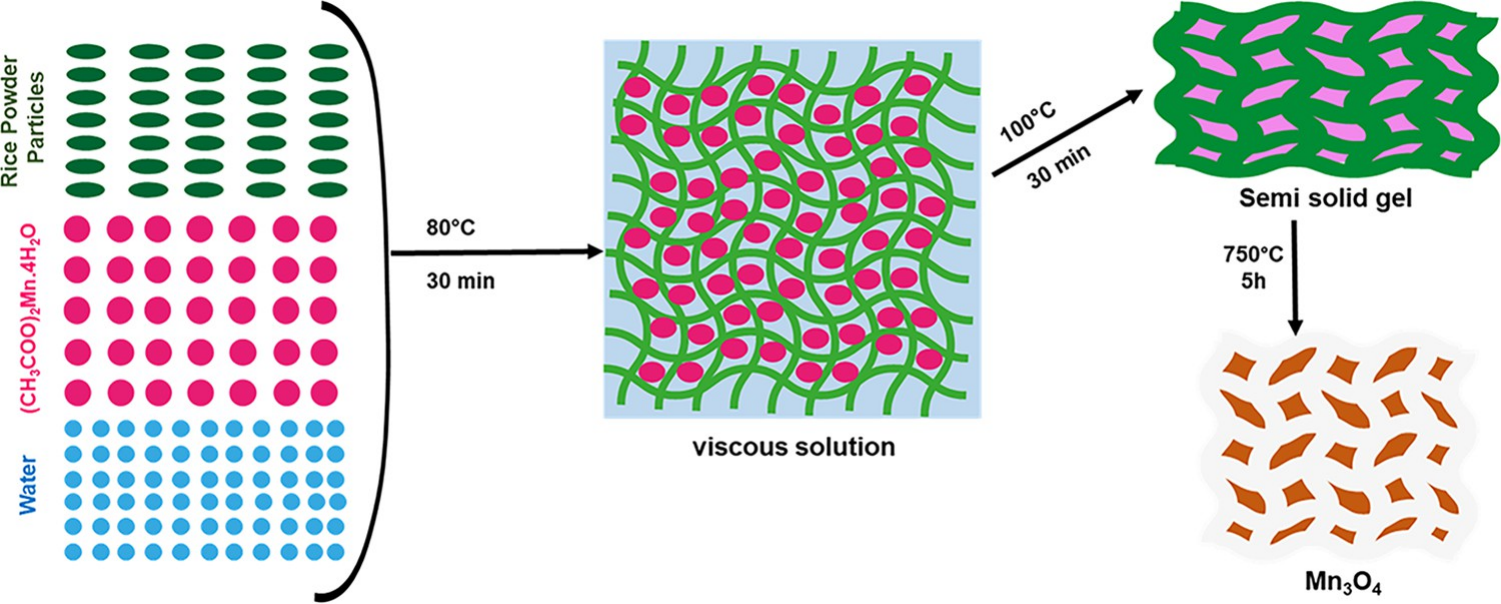
Schematic diagram for the synthesis of Mn_3_O_4_.


20 mL of deionized water was taken in a crucible. 1.0 g of manganese (II) acetate tetrahydrate was added in it and stirred until a homogeneous solution is formed. Then 2.0 g of rice powder was added in the mixture. The weight ratio of manganese (II) acetate tetrahydrate and rice powder is 1:2. Rice powder began to absorb the mixture and got swell. Then it was heated at 80°C. Rice powder began absorb manganese (II) acetate tetrahydrate and water and form a thick white viscous solution. The solution was heated with continuous stirring for 30 min until it got more viscous (semi-solid gel) in appearance. Then it was heated at 110°C for 30 min. After that the crucible containing the mixture was calcined at 750°C for 5h and allowed to cool ad room temperature. Meanwhile the manganese (II) acetate tetrahydrate content in the mixture endure a chemical reaction and form Mn_3_O_4_ and left a dark brown colored solid foam of Mn_3_O_4_. Fig 1. Shows the schematic diagram for the synthesis steps of Mn_3_O_4_. Then it was grinded in a manual mortar/pastel and used for further experiments [28]. The formation of Mn_3_O_4_ nanoparticles could be suggested by following mechanism [29].

$${Mn(C_{7}H_{6}O_{2})}_{n}\overset{750{}^{\circ}C}{\to }{CO}_{2}+H_{2}O+MnO;\;n=1\;or\;2$$

$$2MnO+\frac{1}{2}O_{2}\to {Mn}_{2}O_{3}$$

$${Mn}_{2}O_{3}+MnO\to {Mn}_{3}O_{4}$$

### Coin cell fabrication

After Mn_3_O_4_ was prepared, it was mixed with C-black, PVDF (8:1:1) and NMP as solvent. It was pasted by a mortar/pastel to make paste. Then it was applied on a SS foil to form a thin film on it. Then it was dried at 70°C until it hardened and then cut in to desired shape by battery dice cutting machine to have the final cathode part of CR-2032 coil cell. The pasting and casting of cathode material was manually done. That’s why every battery has different amount of cathode material as well as active material having constant ratio of Mn_3_O_4_, C-black and PVDF was 8:1:1. The amount of active material was calculated after cutting the cathode followed by weighing manually. The battery used for CV and BCD tests had cathode material of 0.02 and 0.008 g respectively. To fabricate a CR-2032 coil cell, the metal cases, cathode, separator, anode, spacer and spring were arranged. Then it was pressed by crimping machine to have the desire final CR-2032 coin cell. The separator was soaked with electrolyte (Aqueous solution of 2.0 M of Zinc sulphate heptahydrate (ZnSO_4_.7H_2_O). About 10 batteries were fabricated and used for electrochemical assessments [30].

## Results and discussion

**Fig 2:**
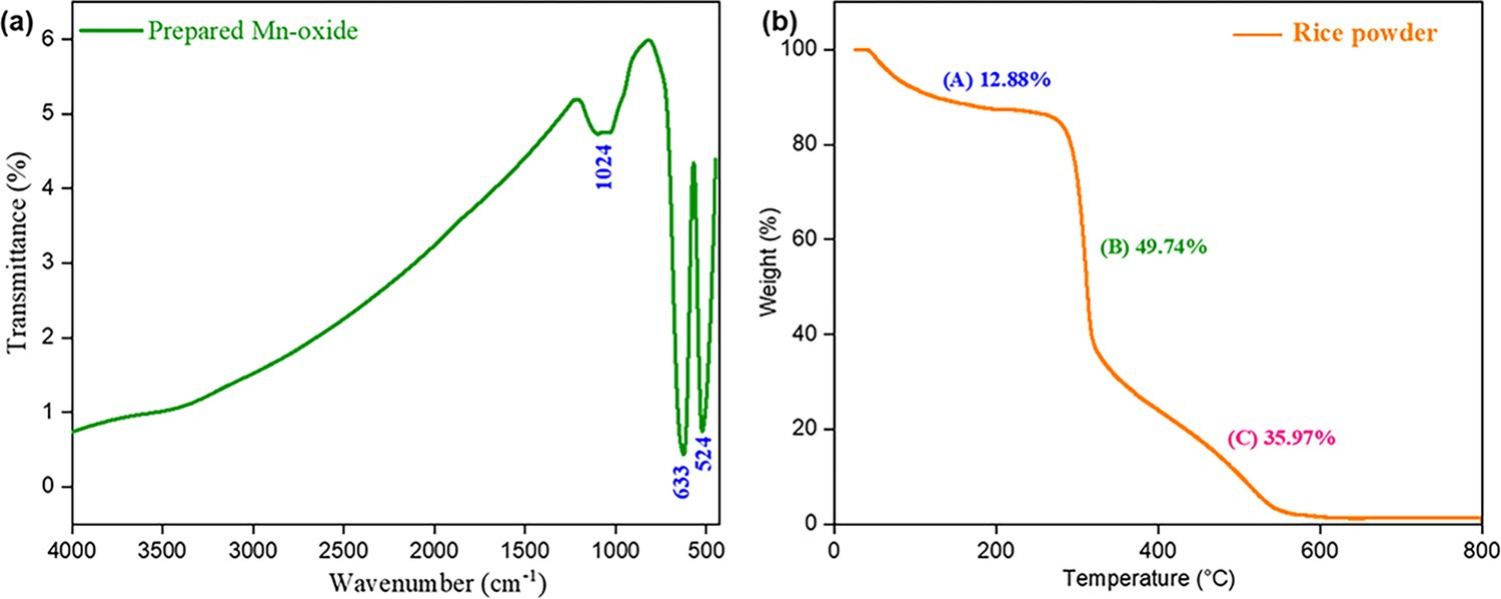
(a) FTIR spectrum of Prepared Mn_3_O_4_ nanoparticle, (b) TGA of rice powder.

Prepared Mn-oxide was analyzed by FTIR spectroscopy (range 450–4000 cm^-1^). The spectrum Fig 2(A) of the prepared Mn-oxide displayed two significant peaks within the range of 450–650 cm^-1^. The peak located at 633 cm^-1^ represents Mn-O stretching vibration in tetrahedral sites, whereas, the peak located at 524 cm^-1^ denotes the distortion vibration of Mn-O in an octahedral site. The weak band at 1049 cm^-1^ could be count for υMn–O–H vibration. This result indicated that the prepared compound was Mn-oxide [31, 32]. FTIR spectrum also consistent with reported references of [33] Mn_3_O_4_ prepared by one step synthesis from Manganese acetate tetrahydrate and N, N-Dimethyl formamide.

The thermal property of rice powder provides the evidence on the physical properties of the components present in it. As a complex mixture, rice powder, consists starch, non-starch, lipids, polysaccharides, sugars, proteins, minerals and fibers at high content [34]. Fig 2(B) shows the TGA of rice powder. The results indicated that, rice powder started to loss its weight at 40.85°C and it loosed 98.59% of its mass at a temperature bellow 100°C, the weight loss was for the moisture content present in it. (A) 40.85°C to 241.31°C associated with water loss; (B) 241.31°C to 322.11°C related with the decomposition of starch, non-starch polysaccharides, sugars, proteins and small amount of lipids.; and (C) 322.11°C to 607.57°C regarding the decomposition of fibers and long chain lipid fragments present in it. After 650°C, about 1.4% weight of rice powder was present which is expected to be carbon [34]. For template assisted synthesis of inorganic nanoparticles, it is necessary to eliminate the template after nanoparticle production to have the pure form of nanoparticles as much as possible. Thus, template could participate in nanoparticle production but could not get itself involve in the application of that nanoparticles. So, as a template at temperature 750°C, rice powder is a very good choice [35].

**Fig 3:**
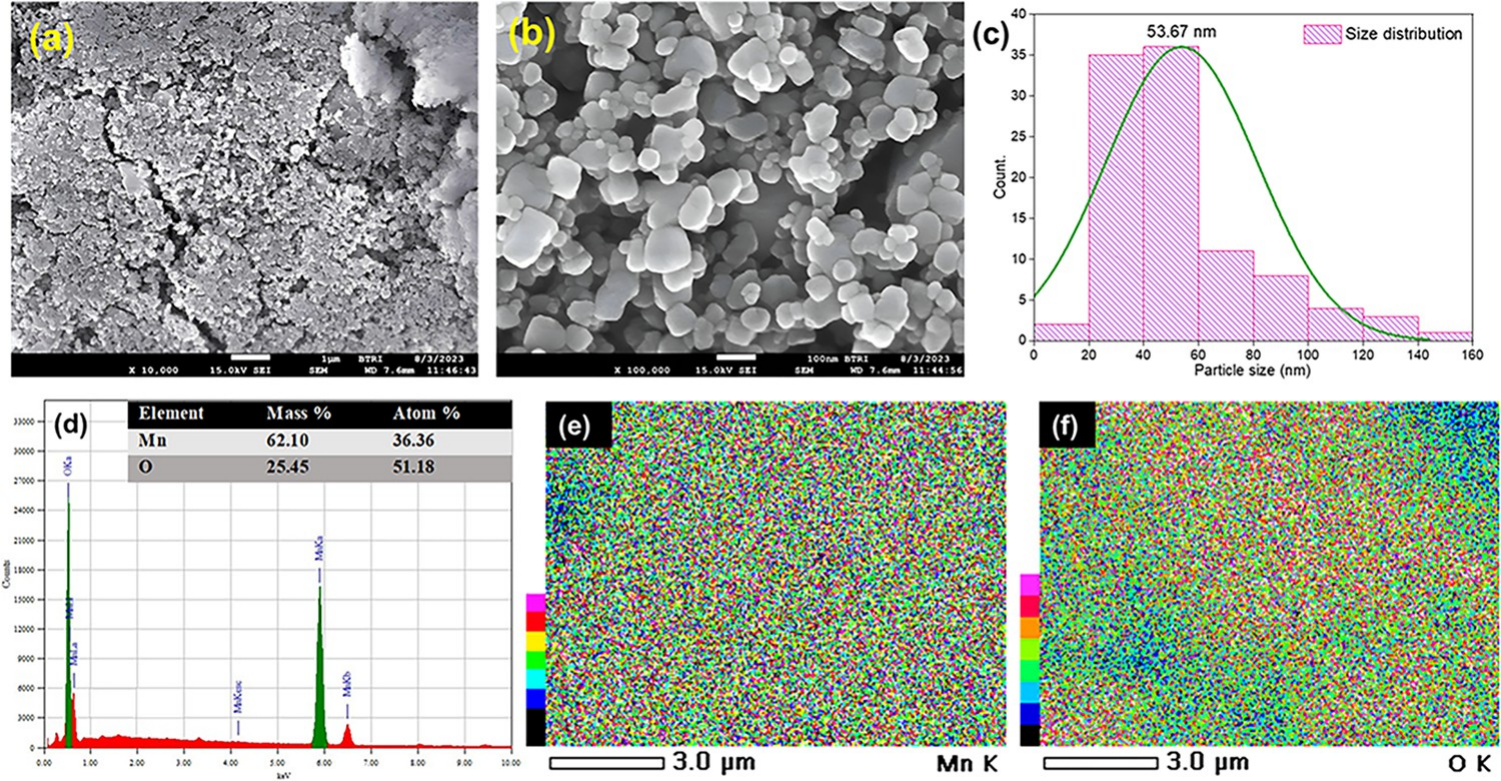
(a and b) FESEM image of prepared Mn_3_O_4_ particles of scaling 1μm and 100 nm respectively, (c) histogram of the particle size distribution of prepared Mn_3_O_4_, (d) EDX spectra of Mn_3_O_4_ particles (inset shows the percentage of major elements), and (e and f) Elemental mapping of Mn and O.

To examine the surface morphology FESEM micrographs were analyzed [Fig 3(A) and 3(B)]. The grains of prepared Mn-oxide were of uneven shapes and sizes. The surfaces were seem to be porous as well. In the calcination process for template elimination at 750°C, the removal of rice powder leads to the existence of pores in Mn_3_O_4_ samples, however these pores are also of irregular sizes. These pores reinforce the electrochemical activity which in this case in favor of energy storage application. Employing ImageJ software, the average particle size was computed by histogram [Fig 3(C)], and it was found to be 53.67 nm [36, 37].

EDX technique was used to assess the composition and stoichiometry of prepared Mn-oxide. Fig 3(D) shows the EDX spectrum of prepared Mn_3_O_4_ samples. Peaks in EDX spectra indicates the presence of Mn and O elements with very little amount of impurity. The EDX lines observed at 5.905 and 0.532 keV are associated for K lines of the Mn and O elements, respectively. The elemental mapping [Fig 3(E) and 3(F)] depicts the elements Mn and O are uniformly distributed over entire Mn-oxide samples. Accordingly, the composition of Mn and O in the prepared sample was 36.36% and 51.18%, respectively, by mass. This indicates the ratio of Mn:O is very close to 3:4. Therefore, from EDX result, it was confirmed that the prepared sample was Mn_3_O_4_ [32].

**Fig 4:**
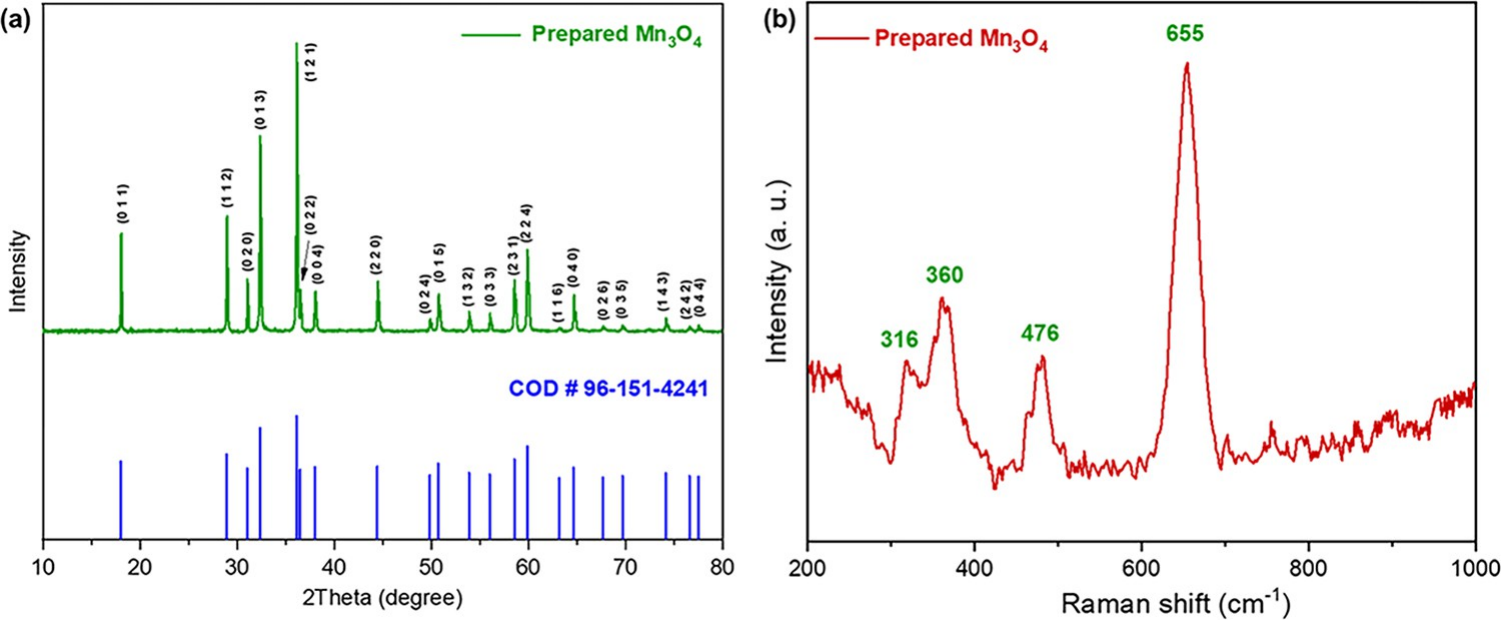
(a) Powder XRD pattern and (b) Raman spectra of the prepared Mn_3_O_4_ nanoparticles.

The prepared Mn-oxide powder was examined by Powder X-ray Diffractometer to identify its phase and crystal structure. Fig 4(A) shows the powder XRD spectrum of prepared Mn-oxide which was happened to be Mn_3_O_4_ nanoparticles. The production of Mn_3_O_4_ was confirmed by the diffraction peaks at 2θ = 18.21°, 28.91°, 31.04°, 32.35°, 36.12°, 36.51°, 38.02°, 44.30°, 49.86°, 50.74°, 53.90°, 56.04°, 58.56°, 59.89°, 63.14°, 64.68°, 67.70, 69.72°, 74.18°, 76.60° and 77.55°; were assigned to the planes (011), (112), (020), (013), (121), (022), (004), (220), (024), (015), (132), (033), (231), (224), (166), (040), (026), (035), (143), (242) and (044), respectively; which were well matched with Crystallographic Open Database (COD) entry #1514240 (pdf # 96-151-4241) [38]. This can be attributed to the tetragonal crystal system of I 41/a m d (141) space group having hausmannite phase of Mn_3_O_4_ nanostructure. No extra peaks were found in XRD pattern for impurities. This denotes the purity of prepared Mn_3_O_4_ powder. This XRD spectrum was well matched with that of Mn_3_O_4_ nanoparticles synthesized by co-precipitation method using manganese chloride and manganese acetate as precursor [39].

The average crystal size was calculated using Scherer equation [40],

$$d=\frac{K\lambda }{\beta Cos\theta }$$ (i)

where, D = crystallite size in nm, K = Scherer constant, λ = wavelength of X-ray in nm, β = full wave half maximum (FWHM) of the XRD peaks and θ is Braggs angle. An average crystallite size of 16.43 nm was observed. The crystallinity was found to be 68.76% [32].

Bragg’s law [40] was used to calculate the d-spacing-

nλ=2dsinθ,,

$$or,\;d=\frac{n\lambda }{2sin\theta }$$ (ii)

Where, λ = 1.5406 Å (wavelength of incident X-ray), θ = Peak position (in radians), n = 1 (order of diffraction), d = interplaner spacing or d-spacing (in Å)

The equation of lattice constant for a tetragonal unit cell was calculated using equation [41],

$$\frac{1}{d^{2}}=\frac{h^{2}+k^{2}}{a^{2}}+\frac{l^{2}}{c^{2}}$$ (iii)

Where, h, k, and l is the miller indices; d indicates interplanar spacing; a and c indicates the lattice constants of unit cell. Using the value of the planes (0 0 4) and (0 2 0) at 2θ = 38.02° and 31.04°, respectively, the lattice parameters were calculated [42]. The values calculated from Eq (ii) and (ii) are given bellow in Table 1. The calculated values of lattice parameter a = b = 5.757644 Å and c = 9. 459276 Å. These values are in very good approximation to the standard values [38].

**Table 1:** Values of peak position, interplanar spacing, miller indices and lattice constants from XRD pattern.

No.	Peak position 2Theta (°)	Interplanar spacing d (Å)	Miller indices (h k l)	Lattice constant			
				a = b		c	
				Standard (Å)	Calculated (Å)	Standard (Å)	Calculated (Å)
1.	18.21	4.867792	0 1 1	5.76	**5.757644**	9.46	**9.459276**
2.	28.91	3.085896	1 1 2				
3.	31.04	2.878822	0 2 0				
4.	32.35	2.765173	0 1 3				
5.	36.12	2.484736	1 2 1				
6.	36.51	2.459084	0 2 2				
7.	38.02	2.364819	0 0 4				
8.	44.30	2.043059	2 2 0				
9.	49.86	1.827474	0 2 4				
10.	50.74	1.797825	0 1 5				
11.	53.90	1.699643	1 3 2				
12.	56.04	1.639705	0 3 3				
13.	58.56	1.575004	2 3 1				
14.	59.89	1.543166	2 2 4				
15.	63.14	1.471330	1 1 6				
16.	64.68	1.439968	0 4 0				
17.	67.70	1.382893	0 2 6				
18.	69.72	1.347684	0 3 5				
19.	74.18	1.277300	1 4 3				
20.	76.60	1.242862	2 4 2				
21.	77.55	1.229993	0 4 4				

Raman spectra of prepared Mn_3_O_4_ is shown in Fig 4(B). Four peaks were observed; one strong peak at 655 cm^-1^ and three small broad peaks at 316, 360 and 476 cm^-1^. These peaks indicated the skeletal vibration of Mn_3_O_4_. The strongest and sharp peak at 655 cm^-1^ could be assigned to A_1g_ mode, which supported the Mn-O breathing vibration of divalent Mn ions in tetrahedral coordination. This result was in good agreement with the mineral hausmannite, as in both chemically prepared samples and commercial powders [43, 44].

**Fig 5:**
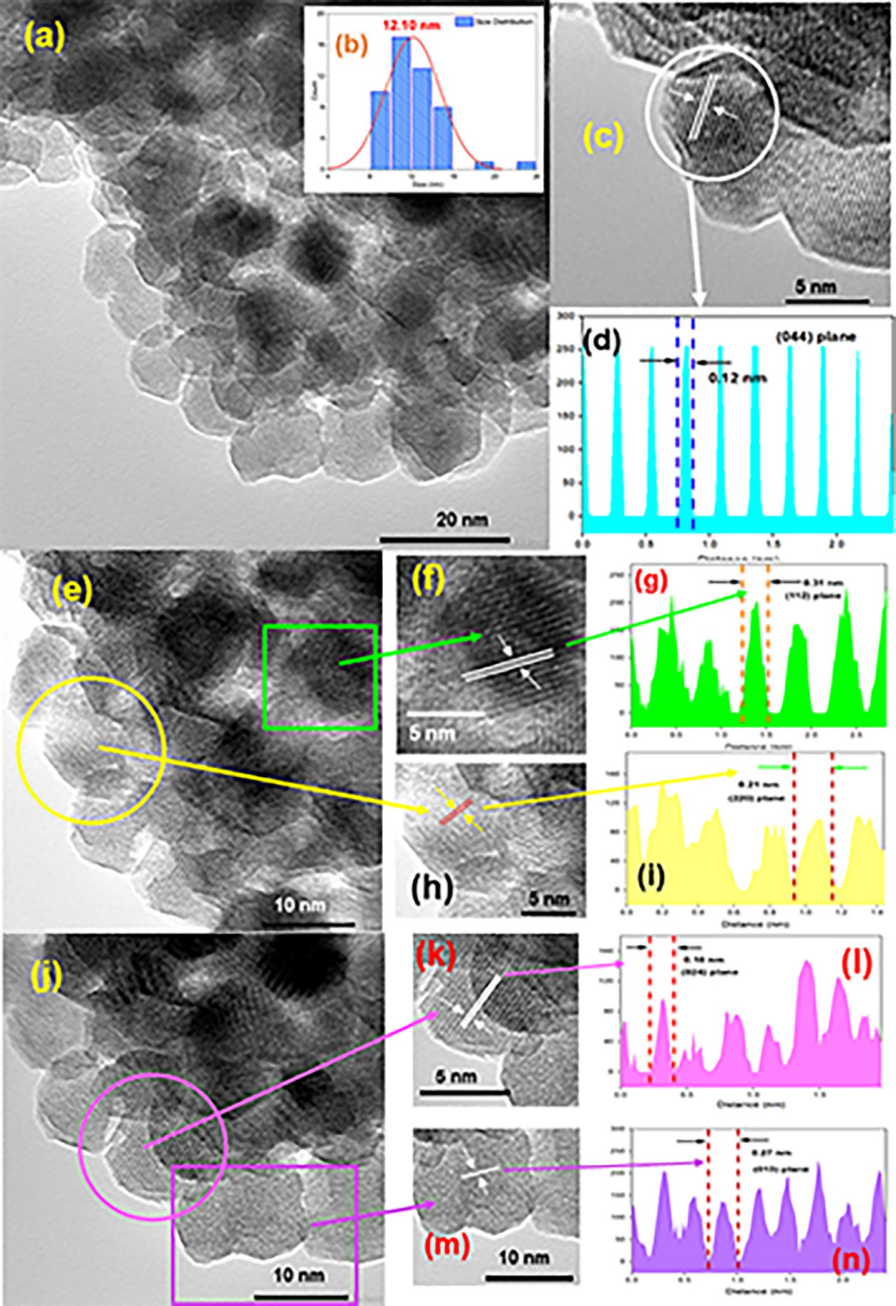
TEM micrographs of prepared Mn_3_O_4_ nanoparticles at scaling (a) 20 nm, (c) 5 nm, (e) & (j) 10 nm; (f) & (h) and (k) & (m) are cross sections of (e) and (j) respectively (inside showing scaling); (d) histogram for particle size distribution of (a); (d), (g), (i), (l) and (n) are corresponding line intensity profile of (c), (f), (h), (k) and (m).

To verify the results of FESEM and XRD, TEM experiment was performed. Fig 5 shows the TEM micrographs of prepared Mn_3_O_4_ nanoparticles. The particles are of irregular shapes and sizes [Fig 5(A), 5(E) and 5(J)]. The average particle size distribution is around 12.10 nm confirmed by histogram [Fig 5(B)]. Highly oriented crystal planes are noticed from Fig 5(C), 5(E) and 5(J) revealing good crystallinity of the Mn_3_O_4_ nanoparticles. ImageJ software was used to find out the d-spacing (0.12, 0.31, 0.21, 0.18 and 0.27 nm) for corresponding planes [(044), (112), (220), (024) and (013)] by creating line intensity graphs [Fig 5(D), 5(G), 5(I), 5(L) and 5(N)] of selected areas [Fig 5(C), 5(F), 5(H), 5(K) and 5(M)]. This values are well matched with Crystallographic Open Database (COD) entry #1514240 (pdf # 96-151-4241) [38].

To analyze the surface properties of prepared Mn_3_O_4_ nanoparticle, BET experiment was carried out. The specific surface area was calculated using classical BET equation [45]-

$$\frac{P}{V(P_{0-P})}=\frac{1}{V_{m}c}x+(\frac{c-1}{V_{m}C})\frac{P}{P_{0}}$$ (iv)

**Fig 6:**
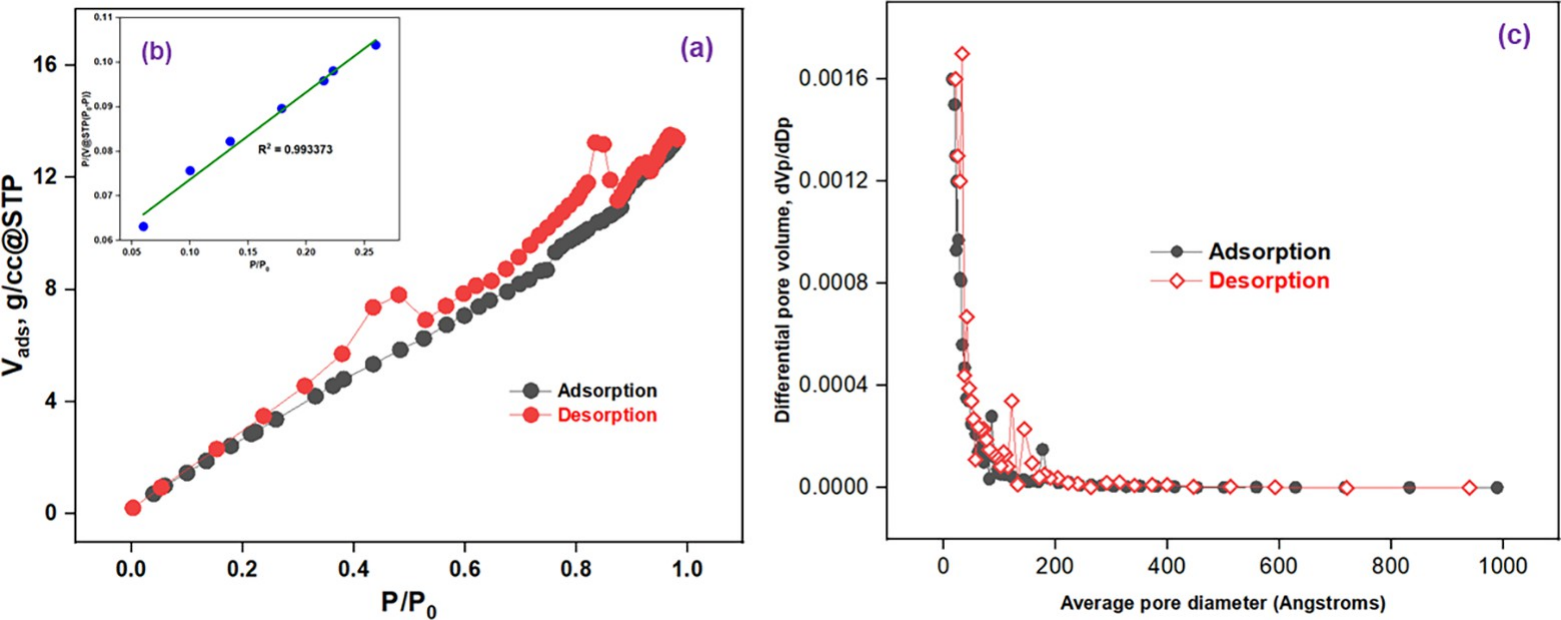
(a) Nitrogen adsorption-desorption isotherm of prepared Mn_3_O_4_ nanoparticle, (b) BET plot, and (c) BJH pore size distribution curves.

Where, V is the volume of adsorbed molecules, V_m_ is the monolayer volume, c is the BET constant related to the adsorbate-adsorbent interaction strength and the heat of adsorption, and P/P_0_ is the relative pressure. The higher value of c, the higher the interaction [46]. Fig 6(A) shows N_2_ adsorption-desorption isotherm of prepared Mn_3_O_4_ nanoparticle. Fig 6(B) shows Brunauer-Emmett-Teller (BET) plot for specific surface area calculation. Here, slope is 0.195866, y-intercept is 0.054145, correlation coefficient, R^2^ is 0.993373. The calculated value of V_m_ is 3.999831 and C is 4.617448. The BET specific surface area (SSA) can be calculated using following equation [45]:

$$SSA=\frac{V_{m}N_{A}a_{m}}{Vm_{s}}$$ (v)

Where, N_A_ is the Avogadro’s number (6.022×10^23^ mol^-1^), a_m_ is the effective cross-section area of one adsorbed molecule of N_2_ (0.162 nm^2^), V is the molar volume of one adsorbed molecule (22.414 L), and m_s_ is the mass of adsorbent. The calculated value of SSA is 17.431304 m^2^/g.

The pore size distribution of prepared Mn_3_O_4_ nanoparticle was determined using Barrett-Joyner-Halenda (BJH) method from adsorption-desorption branch of the isotherm [Fig 6(C)]. Given porosity based on skeletal density of 1.0 g/cc = 0.0202 per gram of sample, the calculated values of average pore diameter and total pore volume is 47.2727 Å and 0.0206 cc/g for both adsorption and desorption studies [47, 48]. This study indicates prepared Mn_3_O_4_ nanoparticle is mesoporous material and is well matched with previously reported results for pure Mn_3_O_4_ nanoparticle [49].

**Fig 7:**
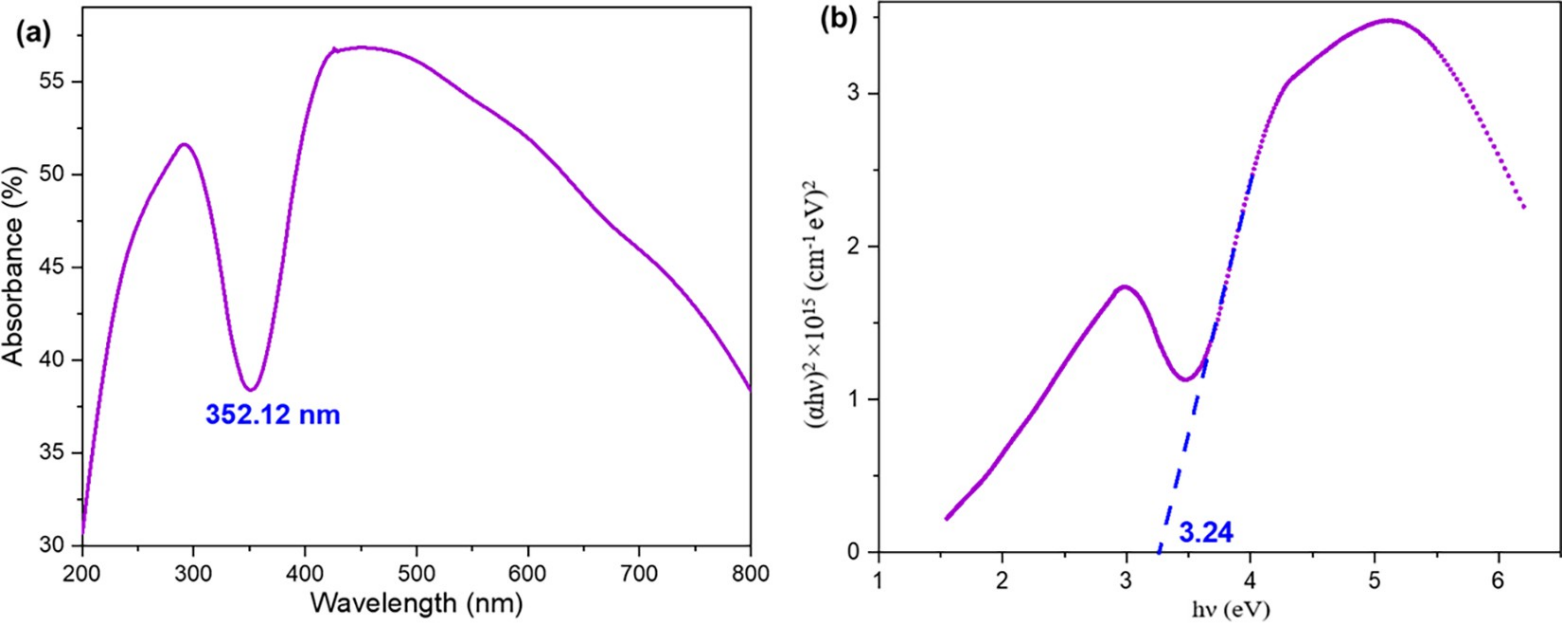
(a) Absorption spectra, (b) Absorption coefficient in the form of (αhν)^2^ versus hν for the prepared Mn_3_O_4_ nanoparticles.

The absorption spectra for the prepared Mn_3_O_4_ nanoparticles is shown in Fig 7(A). The absorption coefficient (α) was calculated from the following equation [50] of optical absorption spectra-

$$\propto =\frac{A}{d}$$ (vi)

Where, d is the thickness of the specimen. The photon dependency of the absorption coefficient can be described by Tauc’s equation [51] given bellow-

$${\left(\propto h\nu \right)}^{2}=B(h\nu -E_{g})$$ (vii)

Where, B is the parameter that depends on the transition probability and Eg is the optical energy gap. Fig 7(B) shows the absorption coefficient in the form of (αhν)^2^ versus hν for the prepared Mn_3_O_4_ nanoparticles. The intercepts of the straight lines with the photon energy axis gives the optical band gap, which was found to be 3.24 eV, represents the semiconductor property of prepared Mn_3_O_4_ nanoparticles. This value is comparably higher than the samples found in the literature [52, 53]. From the part of XRD and TEM, we can see that sample had small crystallite size of highly oriented crystal planes with good crystallinity. In case of metal oxides, sometimes band gap (E_g_) value increases because of the cracking between unit cells when the size of the particle is smaller. This wider band gap of prepared Mn_3_O_4_ nanoparticles occurred because of the quantum confinement effect due to its smaller size of crystallite [29].

**Fig 8:**
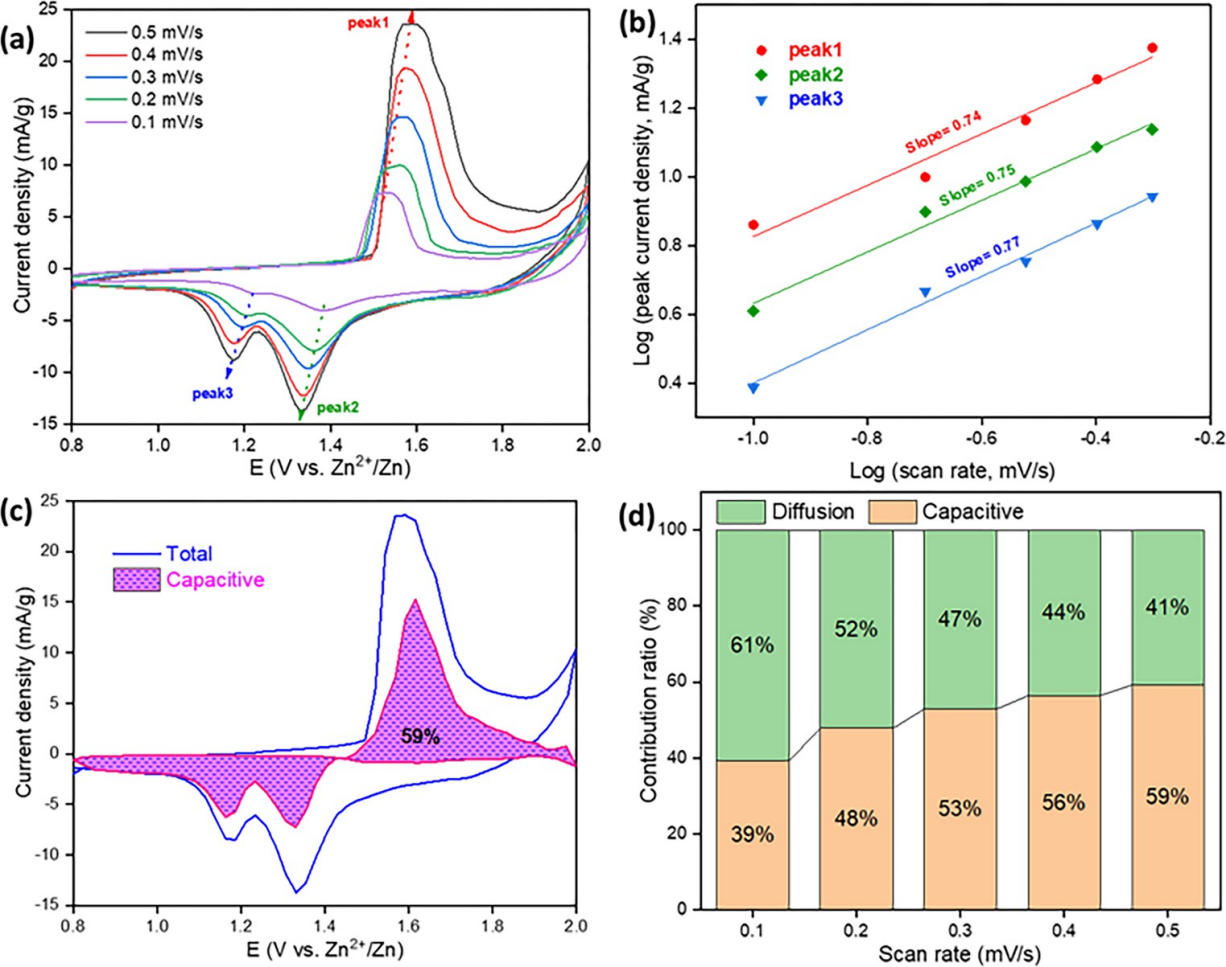
(a) CV curves pf fabricated coin cell at different scan rates, (b) the fitted lines: Log(peak current density) vs Log(scan rate), (c) capacitive contribution to the total capacity at scan rate 0.5 mV/s, and (d) capacitive and diffusion contribution to the total capacity at different scan rates.

CV was performed for the prepared CR-2032 coin cell within electrochemical window +0.8 V to +2.0 V at different scan rates, 0.1, 0.2, 0.3, 0.4 and 0.5 mV/s. one well resolved anodic peak at +1.51V to+1.58 V and two cathodic peaks were appeared at +1.38 to +1.33 V and +1.21 to +1.17 V [Fig 8(A)] and have similar oxidation and reduction peaks in each CV with different scan rates. In anodic process, one sharp oxidation peak at +1.58 V can be attributed to the intercalation of Zn^2+^ ion into the interlayer of hausmannite structure. This indicates the transition of cathode material from Mn_3_O_4_ to Mn_5_O_8_ for Zn^2+^ ion storage purpose. The electrochemical behavior corresponding to the two reduction peaks are the deintercalation of Zn^2+^ into the interlayer of hausmannite structure and H^+^ extraction with the reduction of Mn(II) to Mn(III), respectively. This confirms the process of H^+^ and Zn^2+^ co-deintercalation. With increasing of scan rates the peak heights of peak1, peak2 and peak3, as labelled in Fig 8(A), increased. High scan rate increases the reaction rate thereby quickens the electron transfer process [10]. From this result, probable reaction could be given as-

$$Anode\;reaction:\;Zn↔{Zn}^{2+}+2e^{-}$$

$$Cathode\;reaction:\;2{Mn}_{3}O_{4}+{2e}^{-}↔{Mn}_{5}O_{8}+{Mn}^{2+}$$

$$H_{2}O↔H^{+}+{OH}^{-}$$

Similar result has been found for Mn_3_O_4_ nanoflower based composite cathode for ARZIB [54]. The behavior of the CV was tested by Dunn’s method [55]:

Logi=Loga+bLogv (viii)

Where, a and b are constants. The value of b was obtained between 0.5 and 1.0. If the value of the slope (b) is 0.5, it denotes a diffusion- controlled behavior (Q_d_) and if it is 1.0, it implies capacitive process (Q_c_). A graph was plotted as *Log v* (scan rate) vs *Log i* (peak current density) and the value of slope (b) for anodic (peak1) is 0.74 and cathodic (peak2 and peak3) is 0.75 and 0.77, respectively. From the graph [Fig 8(B)], the result is b = 0.74, 0.75 and 0.77; which is below between 0.5 and 1.0; indicates the total electrochemical storage mechanism includes diffusion controlled pseudo capacitive behavior of a battery characteristics [56, 57]. The storage mechanism of Zn^2+^ has excellent non-diffusion limits because of Mn_3_O_4_ nanoparticles. The capacitive contribution ratio was calculated on the basis of anodic behavior of the cell using the following equation [56]:

$$i_{p}(\nu )=k_{1}\nu +k_{2}\nu^{\frac{1}{2}}$$

$$Or,\;\frac{i_{p}}{v^{\frac{1}{2}}}=k_{1}v^{\frac{1}{2}}+k_{2}$$ (ix)

Where, k_1_ and k_2_ are the slope and intercept of $v^{\frac{1}{2}}$ vs $\frac{i_{p}}{v^{\frac{1}{2}}}$ plot [S1 Fig in S1 File]. The pseudo-charge storage contribution is k_1_ν, and the insertion type capacity is k_2_ν^1/2^ [58, 59]. As compared with the total charge storage contribution, the capacitive contribution was around 59% at 0.5 mV/s scan rate, indicated the process was capacitive dominated [Fig 8(C)]. With the increasing of the scan rates 0.1, 0.2, 0.3, 0.4, and 0.5 mV/s, the capacitive contribution was 39%, 48%, 53%, 56%, and 59%, respectively. The results from Fig 8(D) showed that the capacitive contribution gradually increased with the scan rates and finally reached a maximum value of 59% at 0.5 mV/s. this indicated the enhanced pseudo-capacitive behavior of nanoparticle morphology [60]. In a word, it can be said that the total process was controlled by capacitance at high current densities. These values of b (slope1, 2 and 3) [Fig 8(B)] of CV peaks had very close values as compared to the values with Mn_3_O_4_@C hierarchial nanospheres. Besides the capacitivie contributions are also comparable [10].

**Fig 9:**
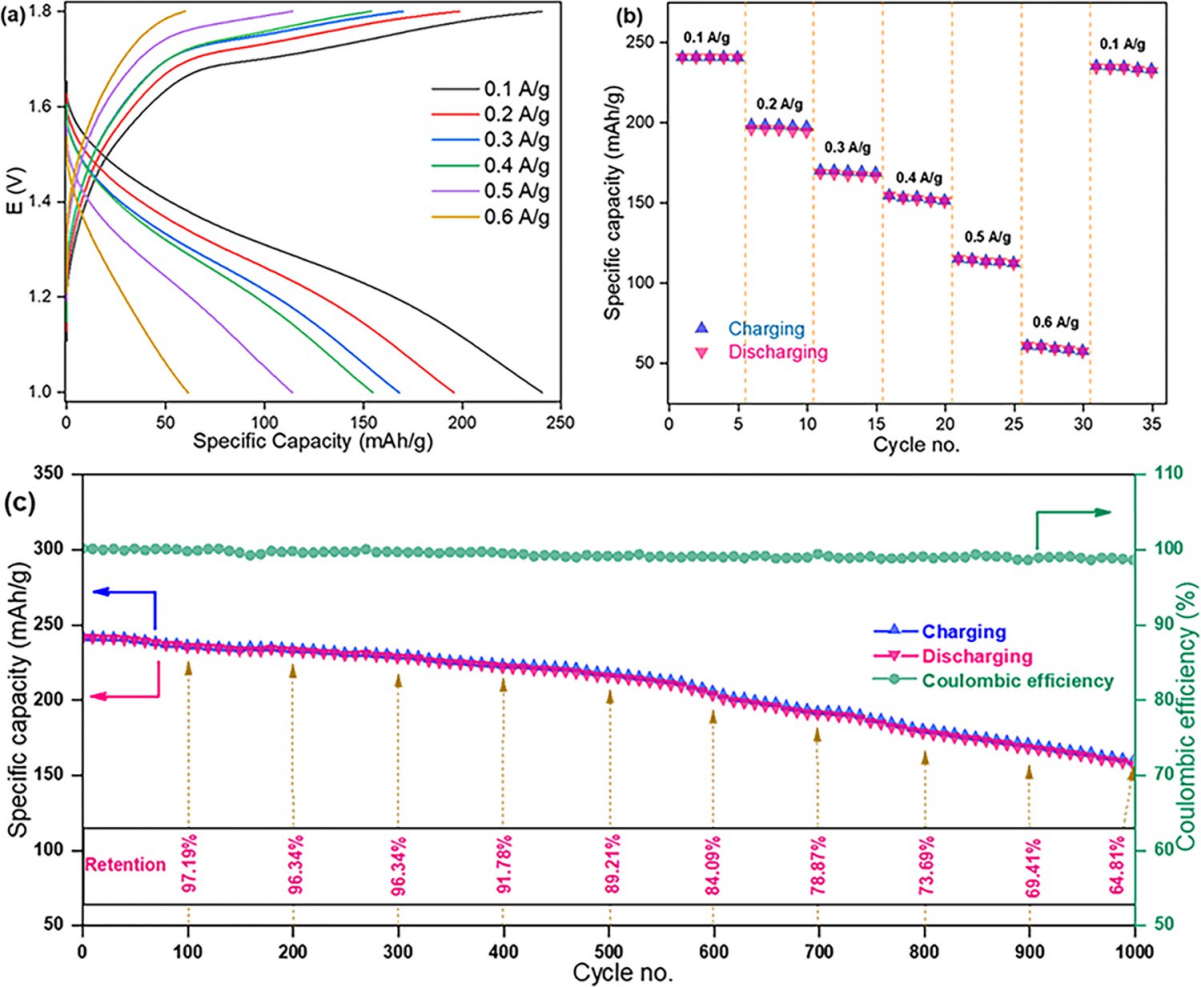
BCD profile of fabricated coin cell (a) Specific capacity at different current density, (b) rate capability at different current density, (c) cycling performance, coulombic efficiency and specific discharge capacity retention at 0.1 A/g over 1000 cycles.

Fig 9(A) represents the battery charge-discharge (BCD) curve of fabricated coin cell having Mn_3_O_4_ as cathode at different current density. It shows with the increasing of current density (0.1, 0.2, 0.3, 0.4, 0.5 and 0.6 A/g) the specific discharge capacity decreases (240.75, 196.15, 168.62, 154.48, 115.04 and 60.71 mAh/g, respectively). Rate capability was also tested at those current densities [Fig 9(B)]. When current density drops back to the initial 0.1 Ag^-1^, the specific discharge capacity was found to be 234.44 mAhg^-1^, which is almost recovered from its initial value 240.75 mAh/g, corresponding to about 97.37% retention. It is seen that the discharge capacity is a litter lower than the charging capacity. This is because after every charging a very small amount of Zn^2+^ may got trapped in the cathode materials or form stable complexes with it, which blocked the available intercalation sites. The coulombic efficiency was found to be 99.98%. BCD cycling performance [Fig 9(C)] was also done at 0.1 A/g current density over 1000 cycles. After 100, 200, 300, 400, 500, 600, 700, 800, 900 and 1000 cycles the specific discharge capacity retention was found to be 97.19%, 96.34%, 94.45%, 91.78%, 89.21%, 84.09%, 78.87%, 73.69%, 69.41% and 64.81%, respectively [61, 62]. These values of specific discharge capacities with retention was very close to the previously reported results of Mn_3_O_4_ applied in ARZIBs [17].

**Fig 10:**
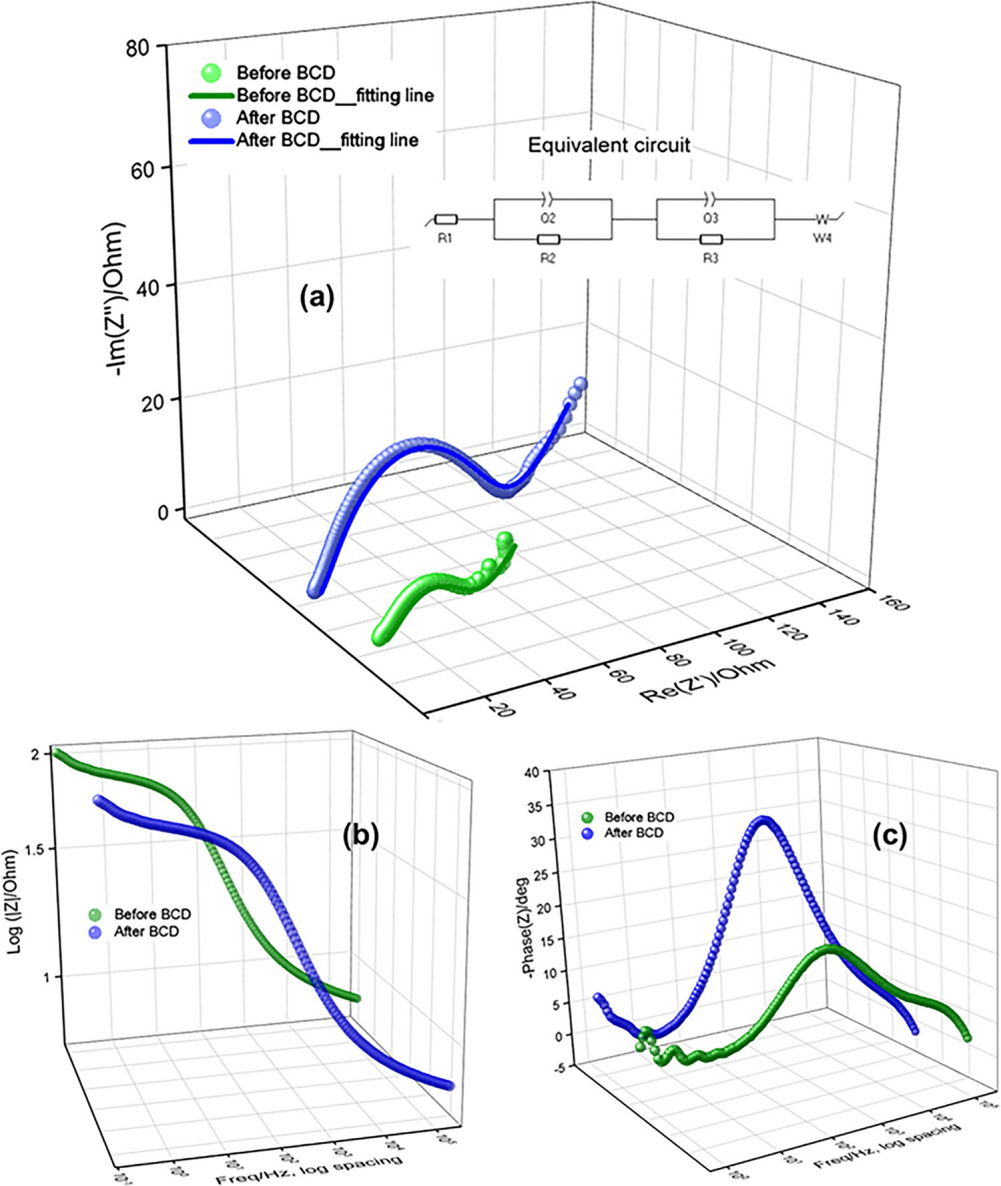
EIS of fabricated CR-2032 coil cell (a) Nyquist plot (inside shows equivalent circuit). Bode plots of (b) frequency dependent impedances and (c) frequency dependent phase angles.

Fig 10 shows the electrochemical impedance profile of fabricated CR-2032 coin cell. The experiment was carried out at potential +1.9 V at 300 kHz to 100 mHz frequency region. This applied potential is a little bit higher than the oxidation potential of the coin cell. The equivalent circuit R1+Q2/R2+Q3/R3+W4 was used to evaluate the Nyquist plot [Fig 10(A)]. Here, R1 represents solution resistance in ohm, R2 represents electrode resistance due to solution electrolyte interface (SEI) contribution in ohm, R3 represents charge transfer resistance in ohm, Q2 and Q3 represents constant phase elements (CPE), which had been usually used for porous materials and W4 represents Warburg impedance in ohm.s^1/2^ [63, 64]. The coin cell showed the values of R1, R2, R3 and W4 were 6.78 Ω, 14.88 Ω, 27.75 Ω and 6.99 Ωs^1/2^ for before BCD; and 7.257 Ω, 32.15 Ω, 38.28 Ω and 16.92 Ωs^1/2^ for after BCD of 1000 cycles, respectively. The resistances are lower for fresh battery but higher after BCD cycling. The equivalent circuit fits at higher frequency and slightly deviates as the frequency decreases. This could be explained in terms of the formation of secondary capacitors with in the coin cell. Furthermore, the coin cell fabrication process was manual; the pasting and casting of cathode material on cathode current collector foil; and the separator was ordinary filter paper. While crimping the cell by crimping machine at high pressure, it may be possible that some of the cathode went into the filter paper. BCD testing took long time also, some cathode materials from the cathode current collector might got detached, traveled through the separator filter paper and went at anode surface, and/or might form a layer on the anode surface, which might be responsible for lower discharge capacity retention. This is also the indication of non-uniform mass transport mechanism in the coin cells [65]. Fig 10(B) shows the frequency dependent impedances of Bode plots. The plots remain at low resistances at high frequency, and show incline lines at low frequency, indicating energy storage capacitive behaviors of the prepared Mn_3_O_4_ nanoparticles. Fig 10(C) shows the frequency dependent phase angles of Bode plots. The highest phase angle is 20.33 to 34.21 degree for before and after BCD of 1000 cycles, show the pseudo capacitive behaviors of Zn^2+^ ion storage at low frequency region [66].

The specific discharge capacities with respect to applied current densities of different Mn-based materials are listed in Table 2. If one emphasize on the simplicity of preparation process and the cost of cathode plate fabrication, this study provides a simple and cost effective technique for a competitive cathode material production for ARZIBs.

**Table 2:** List of specific discharge capacities with respect to applied current densities of Mn-based cathodes in ARZIBs.

Cathode materials	Applied Current Density (mA/g)	Specific Discharge Capacity (mAh/g)	Reference
ε-MnO_2_	0.1	221	[67]
Mn_3_O_4_	0.1	239	[17]
Mn_3_O_4_ nanodot	0.8	182	[68]
α-Mn_2_O_3_	0.2	137	[69]
Mn_3_O_4_@C	0.5	209	[19]
MnCO_3_@ Mn_3_O_4_	0.2	165	[70]
Mn_2_O_3_/ Mn_3_O_4_ composite	1.0	122	[71]
Mn_3_O_4_@N dopped carbon coated carbon cloth	0.2	265	[72]
Mn_3_O_4_	1.0	87	[73]
Mn_3_O_4_@N dopped carbon matrix composite nanorods	0.1	280	[18]
ZnMn_2_O_4_	0.5	150	[74]
MnO_2_/rGO/PANI	0.1	241	[75]
Core-shell Mn_3_O_4_/Carbon (Mn_3_O_4_@C) fiber	0.3	215	[76]
Mn_3_O_4_/GO composite	0.1	215	[77]
β-MnO_2_	0.1	100	[78]
δ-MnO_2_	0.1	126	[78]
α-MnO_2_	0.02	210	[79]
α-MnO_2_	0.05	255	[80]
α-MnO_2_	0.06	302	[81]
α-MnO_2_	0.09	290	[82]
δ-MnO_2_	0.1	269	[83]
Mn_2_O_3_	0.1	150	[84]
**Mn**_**3**_ **O**_**4**_	**0.1**	**240**	**This work**

## Conclusion

ARZIBs show auspicious candidate for secondary energy storage device by safety and environmental compatibility. However, poor cycle stability hinders its progresses. In this work, hausmannite Mn_3_O_4_ nanoparticles were synthesized by a thermal decomposition of Manganese (II) acetate tetrahydrate having rice powder as soft bio-template. Formation of Mn_3_O_4_ nanoparticles were confirmed by a series of spectroscopic and physico-chemical experiments. Fabricated CR-2032 coin cell of ARZIBs using prepared Mn_3_O_4_ nanoparticles as cathode material showed high specific discharge capacity with high columbic efficiency and excellent capacity retention after a long BCD cycling test. This work upholds the improvement of low cost ARZIBs.

## Supporting information

S1 File Supplementary figure. (DOCX)

S1 Graphical abstract (TIF)
