# Supplementary figures and images for: Rice powder template for hausmannite Mn3O4 nanoparticles and its application to aqueous zinc ion battery
Source: PLoS One. 2024 Jun 17;19(6):e0305611. doi: 10.1371/journal.pone.0305611 (PMC11182549; doi:10.1371/journal.pone.0305611)

**Supplementary figure**

**
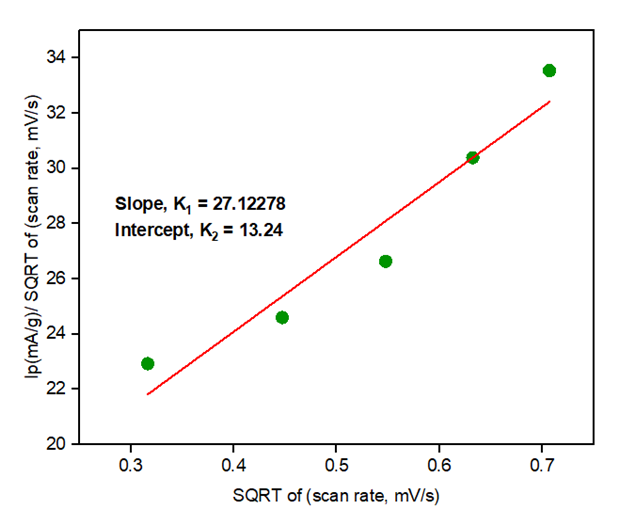
**

**S1 Fig. vs plot of Dunn’s method.**

Supplement: S1 File — (DOCX) [file pone.0305611.s001.docx]

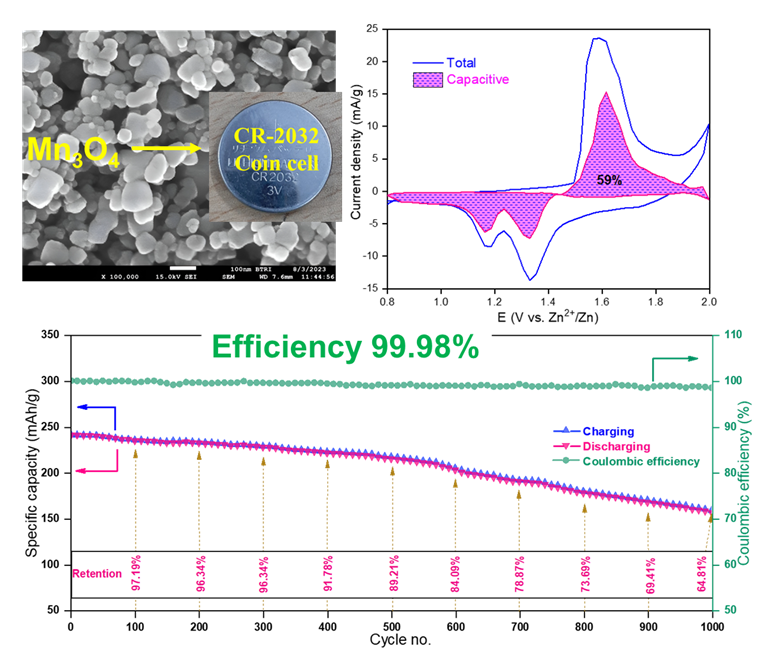

Supplement: S1 Graphical abstract — (TIF) [file pone.0305611.s002.tif]
